# McCune-Albright syndrome

**DOI:** 10.1186/1750-1172-3-12

**Published:** 2008-05-19

**Authors:** Claudia E Dumitrescu, Michael T Collins

**Affiliations:** 1 Skeletal Clinical Studies Unit, Craniofacial and Skeletal Diseases Branch, National Institute of Dental and Craniofacial Research, National Institutes of Health, Department of Health and Human Services, Bethesda, Maryland, USA

## Abstract

McCune-Albright syndrome (MAS) is classically defined by the clinical triad of fibrous dysplasia of bone (FD), *café-au-lait *skin spots, and precocious puberty (PP). It is a rare disease with estimated prevalence between 1/100,000 and 1/1,000,000. FD can involve a single or multiple skeletal sites and presents with a limp and/or pain, and, occasionally, a pathologic fracture. Scoliosis is common and may be progressive. In addition to PP (vaginal bleeding or spotting and development of breast tissue in girls, testicular and penile enlargement and precocious sexual behavior in boys), other hyperfunctioning endocrinopathies may be involved including hyperthyroidism, growth hormone excess, Cushing syndrome, and renal phosphate wasting. *Café-au-lait *spots usually appear in the neonatal period, but it is most often PP or FD that brings the child to medical attention. Renal involvement is seen in approximately 50% of the patients with MAS. The disease results from somatic mutations of the *GNAS *gene, specifically mutations in the cAMP regulating protein, G_s _alpha. The extent of the disease is determined by the proliferation, migration and survival of the cell in which the mutation spontaneously occurs during embryonic development. Diagnosis of MAS is usually established on clinical grounds. Plain radiographs are often sufficient to make the diagnosis of FD and biopsy of FD lesions can confirm the diagnosis. The evaluation of patients with MAS should be guided by knowledge of the spectrum of tissues that may be involved, with specific testing for each. Genetic testing is possible, but is not routinely available. Genetic counseling, however, should be offered. Differential diagnoses include neurofibromatosis, osteofibrous dysplasia, non-ossifying fibromas, idiopathic central precocious puberty, and ovarian neoplasm. Treatment is dictated by the tissues affected, and the extent to which they are affected. Generally, some form of surgical intervention is recommended. Bisphosphonates are frequently used in the treatment of FD. Strengthening exercises are recommended to help maintaining the musculature around the FD bone and minimize the risk for fracture. Treatment of all endocrinopathies is required. Malignancies associated with MAS are distinctly rare occurrences. Malignant transformation of FD lesions occurs in probably less than 1% of the cases of MAS.

## Definition

Originally, the McCune-Albright syndrome (MAS) was defined by the triad of polyostotic fibrous dysplasia of bone (FD), *café-au-lait *skin pigmentation, and precocious puberty (PP) [1,2]. It was later recognized that other endocrinopathies, including hyperthyroidism (reviewed in [3]), growth hormone (GH) excess [4,5], renal phosphate wasting with or without rickets/osteomalacia [6] and Cushing syndrome could be found in association with the original triad [7-9]. Rarely, other organ systems may be involved (liver, cardiac, parathyroid, pancreas) [10].

While MAS is rare, FD is not. FD can involve a single skeletal site (monostotic FD, MFD), or multiple sites (polyostotic FD, PFD) [11-14]. Very rarely PP can be found in association with *café-au-lait *skin pigmentation in the absence of FD (about 1% of the cases), but in general, FD seems to be the most common component of MAS. Therefore, a more clinically relevant definition of MAS, broader than the original triad of FD + PP + *café-au-lait *is: MAS = FD + at least one of the typical hyperfunctioning endocrinopathies and/or *café-au-lait *spots, with almost any combination possible [13,15].

## Epidemiology

MAS is a rare disease and reliable data of prevalence are not available (the estimated prevalence ranges between 1/100,000 and 1/1,000,000). In contrast, the skeletal aspect of the disease, FD, especially monostotic disease, is not rare [16]. FD has been reported to account for up to 7% of all benign bone tumors.

## Clinical description

**Figure 1:**
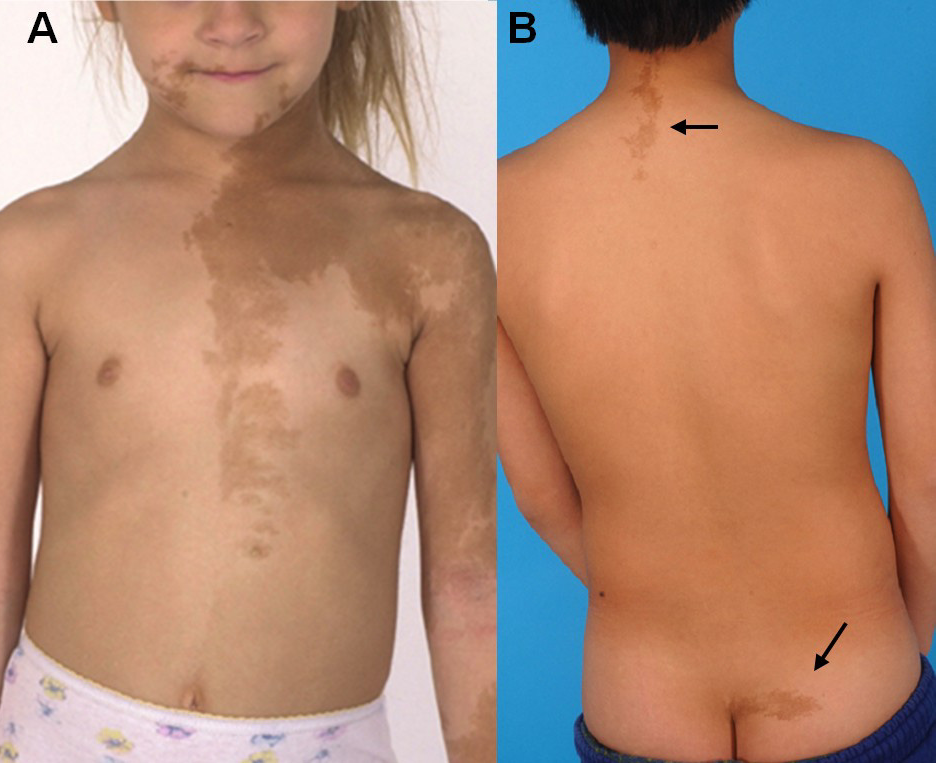
***Café-au-lait skin *pigmentation**. A) A typical lesion on the face, chest, and arm of a 5-year-old girl with McCune-Albright syndrome which demonstrates jagged "coast of Maine" borders, and the tendency for the lesions to both respect the midline and follow the developmental lines of Blashko. B) Typical lesions that are often found on the nape of the neck and crease of the buttocks are shown (arrows).

Typically, the signs and symptoms of either PP or FD usually account for the initial presentation. In girls with PP, it is usually vaginal bleeding or spotting, accompanied by development of breast tissue, usually without the development of pubic hair. In boys, it can be bilateral (or unilateral) testicular enlargement with penile enlargement, scrotal rugae, body odor, pubic and axillary hair, and precocious sexual behavior. In retrospect, *café-au-lait *spots (Fig. 1), which are usually present at birth or shortly thereafter, are the most common but unappreciated "presenting" sign.

**Figure 2:**
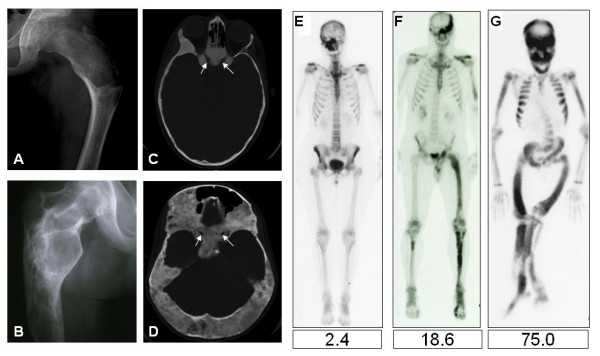
**Radiographic appearance of fibrous dysplasia (FD)**. A) A proximal femur with typical ground glass appearance and shepherd's crook deformity in a 10-year-old child is shown. B) The appearance of FD in the femur of an untreated 40-year-old man demonstrates the tendency for FD to appear more sclerotic with time C) The typical ground glass appearance of FD in the craniofacial region on a CT image of a 10-year-old child is shown. The white arrows indicate the optic nerves, which are typically encased with FD. D) A CT image in a 40-year-old woman demonstrates the typical appearance of craniofacial FD in an older person, with mixed solid and "cystic" lesions. The Hounsfield Unit measurements of "cystic" lesions are quite useful in distinguishing soft tissue "cystic" lesions from true fluid-filled cysts, which are much more uncommon and tend to behave aggressively with rapid expansion and compression of vital structures. E-G) Bone Scintigraphy in FD. Representative ^99^Tc-MDP bone scans which show tracer uptake at affected skeletal sites, and the associated skeletal disease burden score (see ref. Collins, 2005) are shown. E) A 50-year-old woman with monostotic FD confined to a single focus involving contiguous bones in the craniofacial region. F) A 42-year-old man with polyostotic FD shows the tendency for FD to be predominantly (but not exclusively) unilateral, and to involve the skull base and proximal femur. G) A 16-year-old boy with McCune-Albright syndrome and involvement of virtually all skeletal sites (panostotic) is shown [65].

**Figure 3:**
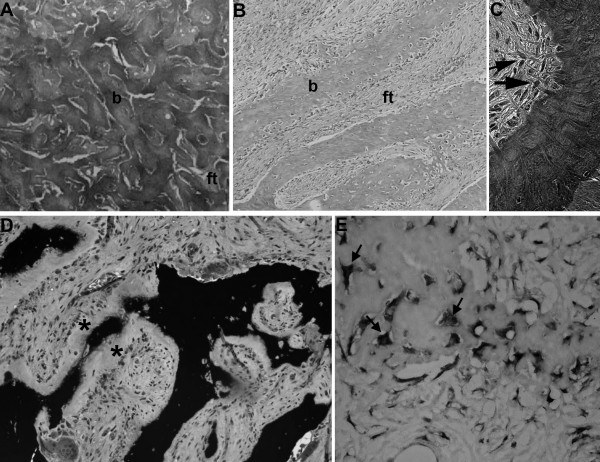
**Representative histological images of FD**. A) Calvarial FD lesions are characterized by uninterrupted networks of bone trabeculae (b) embedded in the fibrous tissue (ft). B) In FD lesions from gnathic bones, newly formed bone trabeculae (b) are deposited within the fibrous tissue (ft) in a typical discontinuous and parallel pattern. C) Collagen fibers perpendicularly oriented to forming bone surfaces (Sharpey fibers, arrows) represent a recurrent histological feature of FD at all skeletal sites. D-E) Osteomalacic changes and FGF23 production in FD. D) In many cases of FD, processing for undecalcified embedding reveals excess osteoid (asterisks) and severe undermineralization of the fibrous dysplastic bone. E) The mineralization defect of the FD tissue is related to elevated levels of FGF23 produced by activated FD osteogenic cells (arrows), as shown by *in situ *hybridization.

**Figure 4:**
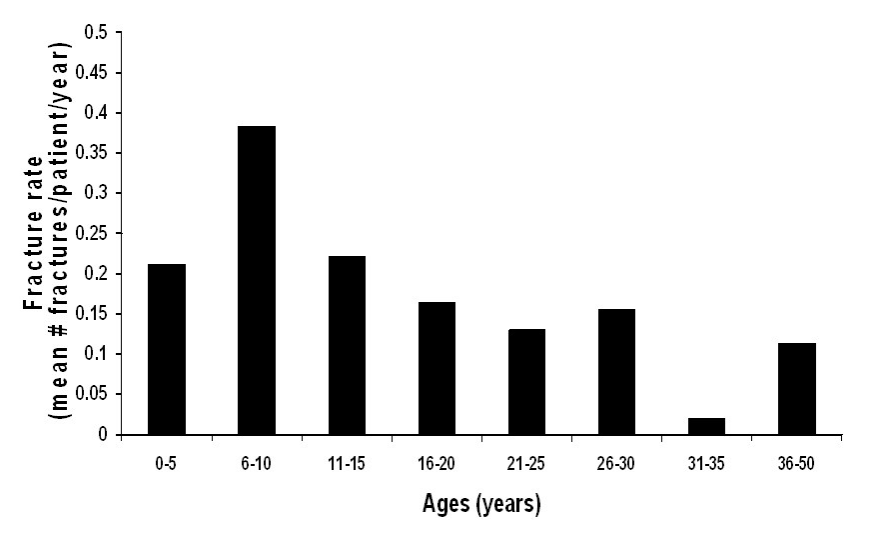
**Fracture rates in FD**. Fractures rates (reported as mean number of fractures per patient per year) are shown. Fractures are more frequent in childhood, with the highest rate occurring between 6–10 years of age. While fractures do lessen after childhood, there is a persistent rate into adulthood [18].

Fibrous dysplasia in the appendicular skeleton usually presents with a limp and/or pain (sometimes reported by children as being "tired"), but occasionally a pathologic fracture may be the presenting sign. Radiographs will demonstrate typical expansile lesions with endosteal scalloping and thinning of the cortex with the matrix of the intramedullary tissue demonstrating a "ground glass" appearance (Fig. 2A &2B). FD in the craniofacial bones usually presents as a painless "lump" or facial asymmetry. Representative radiographic findings and the histological appearance of FD are shown in Figures 2 and 3. The areas most commonly involved are the proximal femora and skull base. The sites of FD involvement are established early; 90% of the total body skeletal disease burden is usually established by age 15 [17]. Hart *et al*. found that lesions in the craniofacial region were established earliest, with 90% of the lesions present by 3.4 years of age. In the extremities, 90% were present by 13.7 yr, and in the axial skeleton, 90% were present by 15.5 yr. The appearance of new lesions later in life is a very uncommon occurrence in FD. The incidence of fractures is greatest in childhood, between the age of 6 and 10 yr, but due to the intrinsic abnormalities in FD bone, some fractures continue to occur into adulthood (Fig. 4)[18].

Other features of the presentation related to the specific aspect of the disease are outlined in Table 1.

**Table 1:** Clinical presentation

**Clinical feature**	**Presentation/Feature**
**Café-au-lait spots**	appearance: "coast-of-Maine", location: nape of neck, base of spine; usually respect midline; trunk & face more commonly involved than limbs (Figure 1)
**Fibrous dysplasia (FD)**	phosphatase, medullary cavity of the bone. [16, 18]
**Craniofacial FD**	nerve decompression is contraindicated [40, 41]
**Axial FD**	usually asymptomatic, scoliosis common and possibly progressive [56] rib pain develops late
**Appendicular FD**	often painful in adults, but pain is quite variable [17, 18]
**Gonadal – Female**	Vaginal bleeding, breast development with little or no pubic hair, secondary central PP often develops, GnRH test [66]
**Gonadal – Male**	common, detectable by ultrasound only, cancer rare, but possible [67]
**Thyroid abnormalities**	ultrasound and biopsy large solid or changing lesions [3], cancer can occur [24]
**Phosphaturia**	worsens pain [6]; etiology is FGF23 of FD bone origin [43]
**Growth hormone (GH) excess**	suggests GH excess, worsens craniofacial FD [5, 41]
**Cushing syndrome**	quite ill, spontaneous resolution possible [9]
**Additional clinical features**	alopecia [70], myxomas and others cutaneous abnormalities [71]

### Malignancies in MAS

While malignancies associated with MAS are distinctly rare occurrences, they warrant mentioning due to their importance. Malignant transformation of FD lesions is probably the most common and best described malignancy that occurs in association with MAS [12,16,19]. This occurs in probably less than 1% of the cases of FD/MAS. High dose external beam radiation is a risk factor for sarcomatous transformation [19]. There may be a greater tendency for malignant transformation to occur in patients who have concomitant GH excess [20]. While some have suggested that sarcomatous transformation of skeletal lesions may occur more commonly in patients with Mazabraud's syndrome (benign intramuscular myxomas in association with long standing FD) [21], this may represent selection bias.

In addition, the risk of breast cancer may be elevated in patients with MAS [22,23]. Besides the published reports, in the series of approximately 120 patients seen at the National Institutes of Health (NIH), the prevalence of breast cancer was approximately 2.5% (n = 3). In this group of three patients, there also seems to be an effect of GH excess, in that all patients who had breast cancer also had GH excess (unpublished data).

Thyroid cancer [24] and testicular cancer (unpublished data) are also rare occurrences.

## Etiology

**Figure 5:**
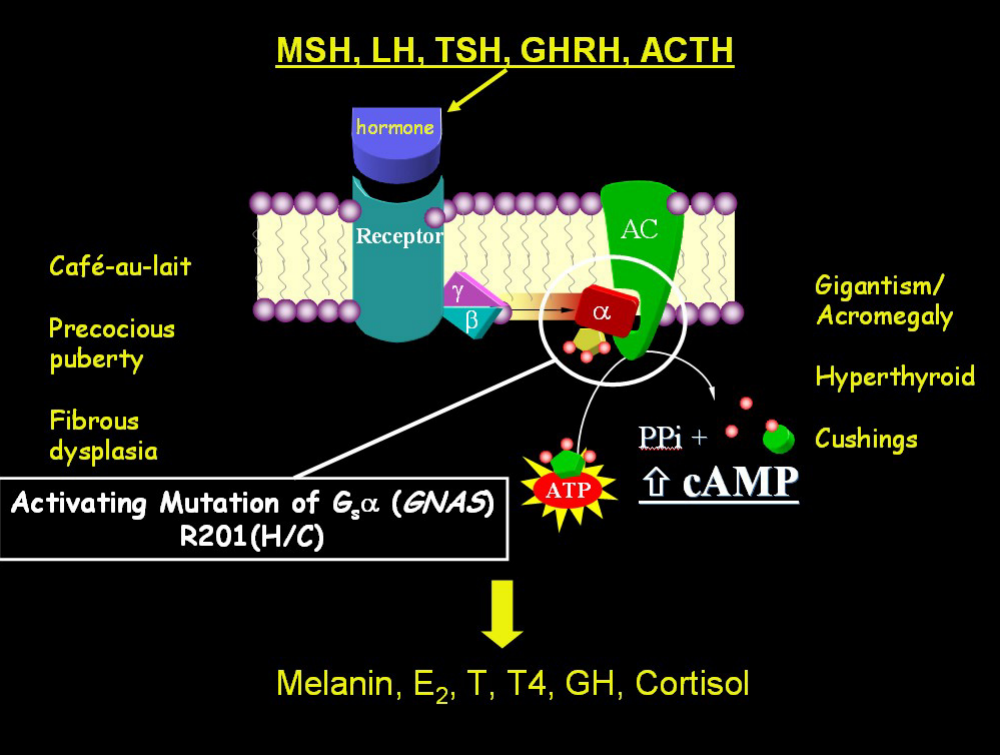
**Molecular defect and phenotype in McCune-Albright syndrome (MAS)**. The hormones MSH (melanocyte stimulating hormone), LH (luteinizing hormone), TSH (thyroid stimulating hormone), GHRH (growth hormone stimulating hormone), and ACTH (adrenocrotical stimulating hormone) all signal through the G protein (alpha, beta, gamma subunits) pathway. In MAS, the alpha subunit is mutated in such a way as to induce constitutive activation of adenylate cyclase, and thus produce high levels of intracellular cAMP. This results in increased production of melanin, estradiol (E_2_), testosterone (T), thyroxine (T4), growth hormone (GH), and cortisol. Dysregulated production of these hormones results in *café-au-lait *spots, precocious puberty, fibrous dysplasia, acromegaly, hyperthyroidism, and Cushing's disease, depending on the tissue harboring the somatic mutation.

The observation that the G protein/cAMP/adenylate cyclase signaling pathway was central to all of the tissues involved in MAS eventually led to the discovery that mutations in the regulatory G_s_**α **protein (encoded by the *GNAS *gene) were the underlying molecular etiology of MAS [25,26] (Fig. 5). In all published cases of MAS, PFD, and even MFD, activating mutations of G_s_**α **at the R201 position have been identified [27]. More recently, mutations at the Q227 position have been found in association with FD [28].

**Figure 6:**
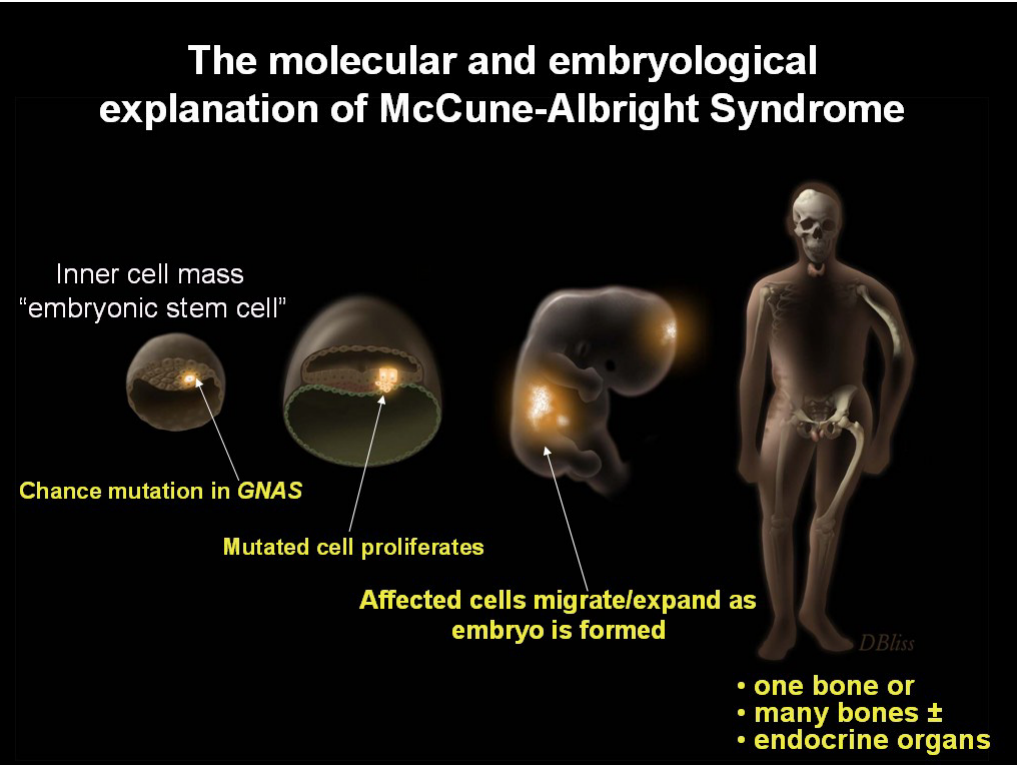
**Molecular and developmental defect in McCune-Albright syndrome (MAS)**. A sporadic mutation occurs in a single cell (bright spot) at some point early in development. If this occurs at the inner call mass stage (embryonic stem cell stage), tissues from all 3 germ layers will be affected. As the cells derived from this mutated clone are dispersed throughout the organism, the final phenotype emerges, MAS.

The lack of vertical transmission of the disease, along with the observation that skin and bone lesions tend to respect the midline and be on one or the other side of the body, has led to the unproven, but accepted, concept that the disease is the result of postzygotic mutations, and that patients are therefore somatic mosaics. The point in time in development at which the mutation occurs, the specific cell in which it occurs, and to where its progeny migrate, determines what tissues will be affected, and thus the phenotype. Therefore, in cases in which tissues of endodermal, mesodermal, and ectodermal origin are involved, it would appear that the mutation occurred at the inner cell mass stage (Fig. 6) [29].

## Diagnosis, diagnostic criteria, diagnostic methods, differential diagnosis

Diagnosis of MAS is usually established on clinical grounds. Plain radiographs are often sufficient to make the diagnosis of FD (Fig. 2). Isotopic bone scans are the most sensitive tool for detecting the presence of FD lesions, and are often useful, especially at the initial evaluation, for determining the extent of the disease and predicting functional outcome (Fig. 4E,F, &4G) [17,30]. FD has a typical appearance on radiographs described as "ground glass." In general, lesions in the long bones have a "lytic" appearance. The lesions usually arise in the medullary cavity and expand outward replacing normal bone, which results in thinning of the cortex (Fig. 2A &2B). It is usually the metaphysis and/or the diaphysis that are involved, with sparing of the epiphysis. It is possible for any bone to be involved, but the skull base and the proximal femur are the sites most commonly involved [16,31,32]. Due to the fact that these lesions are undermineralized [27], the bones are "soft" and prone to deformation, as exemplified by the classic "shepherd's crook" deformity of the proximal femur (Fig. 2A).

**Figure 7:**
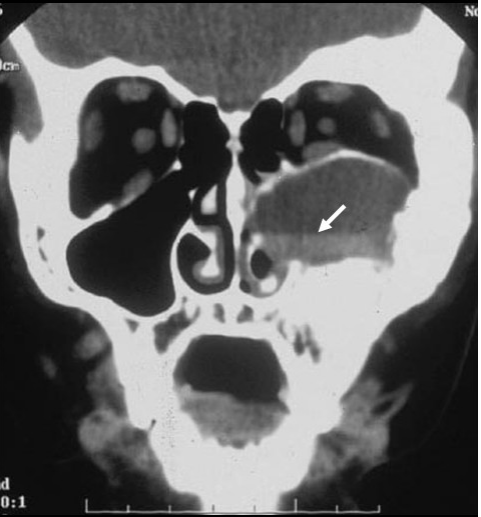
**Fluid-filled cyst in FD**. True fluid-filled cysts can occur in FD lesions. Shown is the CT scan of fluid-filled cyst that arose in a 12 year old boy with MAS who presented with facial nerve parasthesias and displacement of the orbit that occurred over approximately one week. The fluid-filled nature of the lesion is demonstrated by the fluid-fluid level (arrow). These lesions are more frequent in the craniofacial bones and can be aggressive. They usually require prompt surgical intervention.

FD in the craniofacial bones tends to have a "sclerotic" appearance on plain radiographs. This is due to the relatively greater degree of mineralization of FD tissue in the craniofacial bones (Fig. 3) [33]. Computed tomography (CT) scanning is the best technique for imaging FD lesions in the skull, revealing a "ground glass" appearance. In children and young adults, the lesions appear homogeneous on CT, but in older patients the appearance is mixed, with the development of "cystic" lesions in some areas. The density of these areas is that of soft tissue, so while they may have a cystic appearance they are not true cysts. That said, it is possible for true cysts to develop in FD, both in the long bones, but more often in the craniofacial bones (Fig. 7). This has occurred in about 5% of the patients with FD in the NIH cohort (unpublished data). If needed, bone cysts may be diagnosed using magnetic resonance imaging (MRI). The cysts tend to have a more aggressive course. They can expand rapidly and produce symptoms which vary, depending on the location. One of the complications can be the fracture through a cyst. This usually requires surgical intervention.

Biopsy of FD lesions can confirm the diagnosis if doubt remains after review of the radiographs. One characteristic of FD bone is the absence of the lamellation pattern seen in normal bone under polarized light. This indicates that the matrix produced in the lesions is of the woven type. The histopathological description of FD is often described as a "Chinese writing" pattern, and with special preparation and stains used to detect mineralized and unmineralized tissue, extensive areas of unmineralized osteoid are evident (Fig. 3) [27]. For an extensive description of the histopathological changes that can be observed in FD, the reader is referred to Riminucci *et al*. and Corsi *et al*. [33,34].

### Genetic testing

Genetic testing is possible, but is not routinely available (see below). Because of the somatic mosaic nature of the disease, a negative result from readily available (but unaffected) tissue does not exclude the presence of the mutation. Testing on leukocyte DNA is possible [35], but it is unreliable. In most cases, genetic testing contributes little to the diagnosis and not at all to management. There is no known genotype/phenotype correlation, so knowledge of the specific mutation does not affect management. For this reason, when the diagnosis of MAS is suspected or established, it is important to be cognizant of the spectrum of tissues that possibly can be involved, and to screen for involvement. Screening, at a minimum, requires a medical history and physical examination, and usually involves specific imaging and biochemical testing.

The following are listed as potential sources for genetic testing: Center for Genetic Testing at Saint Francis [36], Genome Diagnostics [37]. In addition, the research laboratories of Professors Francis Glorieux, Shriners Hospital for Children (Montreal, Canada), Paolo Bianco (Rome, Italy) and Charles Sultan (Montpellier, France) can perform genetic testing on a research basis.

### Differential diagnosis

MAS is most commonly confused with neurofibromatosis (NF), usually when a child presents with a large *café-au-lait *spot. The location and shape of the spots usually can help to distinguish between the MAS and NF. The spots in MAS have jagged borders (coast of Maine), whereas those in NF are smooth (coast of California). Although the spots can cross the midline, more often, they demonstrate a "respect" of the midline. Frequent locations are the nape of the neck and the crease at the apex of the buttocks (Fig. 1). In MAS, the skeletal disease (PFD) almost always involves one or both proximal femurs and/or the skull base, as well as other locations. Skeletal involvement in NF is uncommon and usually involves the diaphyses of the long bones, especially the tibiae, often leading to pseudoarthrosis [38,39].

When precocious puberty is the presenting sign, the differential diagnosis includes idiopathic central precocious puberty, and an ovarian neoplasm. Suppressed gonadotropins exclude central PP. Other components of the MAS (skin pigmentation, skeletal changes on x-ray or bone scan, *etc*.) can help to assure the clinician that the ovarian cyst is not neoplastic.

Isolated skeletal lesions in the absence of skin or endocrine findings represent FD (MFD or PFD). Osteofibrous dysplasia (ossifying fibroma of long bones, so-called Campanacci's lesion) may be confused with FD. These lesions are almost exclusively found in the tibia and fibula, and are histologically distinct from FD [12]. Non-ossifying fibromas (NOF) may also share radiological and histological similarities with FD in the long bones. Lack of multiple skeletal foci and absence of extraskeletal findings may help to distinguish FD from NOF. FD in the jaw may share several histological features with cemento ossifying fibromas, which can be confused with FD. The cemento ossifying fibroma lesions tend to behave more aggressively than do FD lesions. Testing for the *GNAS *mutation, if tissues and assays are available, may be helpful in distinguishing cemento ossifying fibroma lesions from FD.

### Specific diagnostic considerations and follow-up

#### A. Skeletal

Skeletal disease, especially involving the skull base, is very common. Vision and/or hearing loss are uncommon, and sarcomatous transformation is rare (<1%). FD in the appendicular skeleton tends to quiet with age, but the course of craniofacial disease is less clear [18,40]. Nuclear medicine bone scans are useful for not only detecting the extent of the disease, but quantifying the skeletal disease burden of FD and predicting functional outcome [30].

#### 1. Craniofacial FD

CT scan of the skull is the most useful test for diagnosis of craniofacial FD (Fig. 4). CT should be repeated periodically, annually then less frequently once stability has been demonstrated. Vision should be evaluated, ideally by a neuro-ophthalmologist initially, then periodically; annually or less frequently once stability has been demonstrated. Hearing evaluation is recommended at baseline to assess involvement and should also be repeated periodically. All endocrinopathies which adversely affect bone should be screened for and treated. GH excess in particular may adversely affect craniofacial FD [5,40,41]. ^99^Tc-methyl diphosphonate (^99^Tc-MDP) bone scan at baseline and periodically is recommended.

#### 2. Axial and appendicular skeleton

**Figure 8:**
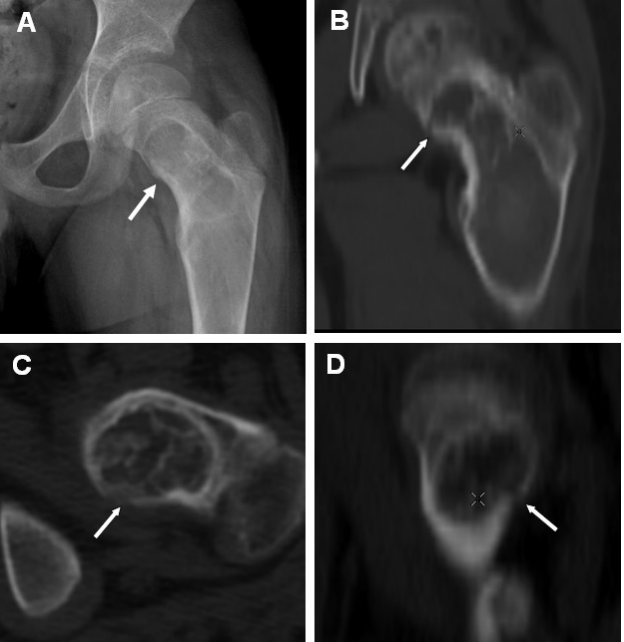
**CT for detecting subtle fractures**. A) This radiograph was from a 9-year-old boy who complained of new-onset, focal pain in the groin. No discernable fracture is apparent. B) Reformatted CT images of the lesion revealed a fracture in the medial proximal femur (arrows).

Plain radiographs, demonstrating the classic ground glass appearance, are usually sufficient for the diagnosis of FD in the axial and appendicular skeleton (the axial skeleton is comprised of bones of the skull, hyoid bone, vertebra, sternum and ribs; the appendicular skeleton includes the hip, pelvic bone and the shoulder girdle (clavicle and scapula) (Fig. 2). However, x-rays are often unable to detect new, small microfractures. When new focal pain develops in an FD lesion and no fracture is evident on plain radiograph, CT and or MRI can be useful for detecting subtle fractures (Fig. 8). The classic lesion of the proximal femur in FD, the shepherd's crook deformity is common (Fig. 2).

### B. Endocrine

#### 1. Gonads

PP is more common in girls then boys. However, small testicular masses of Leydig and/or Sertoli cell hyperplasia are common in boys. These are usually never detected on physically examination. Therefore, ultrasound of the testes is recommended in all males with FD and MAS. It is probably safe to monitor testicular masses without surgical intervention as cancer is probably quite rare. The PP of MAS in girls is due to high levels of serum estradiol due to intermittent autonomous activation of ovarian tissue. Some of them will progress to central PP, secondary to activation of the hypothalamic-pituitary-gonadal (HPG) axis. Since central PP usually develops in children who have had PP for some years, children with PP should be evaluated for secondary central PP.

#### 2. Thyroid

Hyperthyroidism is common (38%) [3]. Evidence of thyroid involvement without frank hyperthyroidism is even more common (63% in the NIH series, unpublished data). This is manifested as a relatively suppressed thyroid stimulating hormone (TSH) with elevated Triiodothyronine (T3+) and an abnormal thyroid gland on ultrasound. Some of these patients may go on to develop frank hyperthyroidism, therefore, follow-up with measurement of TSH, free thyroxine (FT4), T3, and thyroxine (T4), and periodic ultrasound is recommended to detect those patients that progress to frank hyperthyroidism. While the development of thyroid cancer in patients with MAS is rare [24], patients should be monitored for this possibility. This is accomplished by annual ultrasound of the thyroid. The ultrasonographic appearance of the thyroid in MAS is complicated [42]. There are usually admixed areas of relatively normal appearing thyroid with adjacent cystic areas. Suspicion for cancer should be raised if a large or expanding solid lesion is noted. This should prompt fine needle aspiration with cytopathological evaluation.

#### 3. Renal phosphate wasting

Renal phosphate wasting, as part of a proximal tubulopathy, with or without hypophosphatemia, and/or rickets/osteomalacia is common [6]. The etiology is likely due to elaboration of the phosphaturic factor, fibroblast growth factor-23 (FGF23), by FD tissue [43]. Measurement of serum phosphate and calculation of renal phosphate handling by either the tubular reabsorption of phosphate (TRP) or maximal tubular reabsorption of phosphate per glomerular filtration rate (TmP/GFR) are recommended. While it is debatable whether or not hypophosphatemia, *per se*, should be treated, or if treatment should be reserved for only patients with rickets or biopsy-proven rickets [44,45], it is our feeling that frank hypophosphatemia should be treated (see Additional file 1).

#### 4. Pituitary

GH and prolactin excess are common (21%), and the signs and symptoms can be very subtle. Since GH excess can worsen craniofacial bone disease [5,40,41], it is important to make the diagnosis and treat. All patients should have an oral glucose tolerance test (OGTT) to look for non-suppressible GH at least once. Most GH secreting tumors are co-secretors of GH and prolactin, so prolactin levels should also be assessed, as hyperprolactinemia can independently have an adverse affect on gonadal function.

#### 5. Parathyroid

Primary hyperparathyroidism in MAS is rare and is probably not a part of the syndrome [46]. Secondary hyperparathyroidism, usually due to vitamin D deficiency is common in the general population as well as in FD/MAS [47-50]. Hyperparathyroidism can worsen FD and should be treated [34]. Total or ionized serum calcium and parathyroid hormone (PTH) need to be assessed periodically.

#### 6. Adrenal

**Figure 9:**
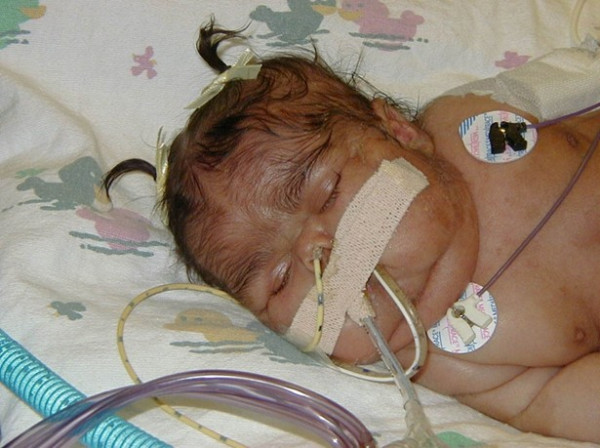
**Appearance of Cushing in a neonate**. Rounded (moon) facies with plethora and facial hirsutism are present. This is usually accompanied by the presence of typical *café-au-lait *spots elsewhere on the examination.

Cushing syndrome can occur in the neonatal period, but has not been reported past the first year. Some cases of neonatal Cushing resolve spontaneously [9]. Cushing syndrome must be considered and screened for in very young patients. The physical examination is usually suggestive and shows moon facies with plethora, hirsutism, and lack of linear growth (Fig. 9). If suspected, laboratory evaluation with some combination of a 24-hour urine for urinary free cortisol (if possible), midnight, or salivary cortisol, and/or dexamethasone suppression test are indicated. Adrenal reserve should be checked in patients in whom Cushing resolved spontaneously (personal comment, in the NIH series 2 out of 9 patients with Cushing had spontaneously cure and inadequate adrenal reserve later in life).

## Genetic counseling

The most important aspect of counseling families is to assure the patients or families that there is no vertical transmission of the disease, nor are there any known environmental associations or predilection for ethnic groups. Therefore, parents need not feel "responsible," and patients can be assured they will not transmit the disease to their offspring.

## Antenatal diagnosis

Not relevant.

## Management, treatment, and prognosis

### A. Skeletal

#### 1. Craniofacial FD

In the vast majority of cases of craniofacial FD, surgery is not needed, and observation is the correct approach. Observation involves annual vision and hearing testing, and imaging. Vision should be checked by a neuro-ophthalmologist. CT of skull and/or mandible is the imaging test of choice for the cranial structures in general, but MRI will give better resolution of the optic nerves. Cranial nerves are often surrounded by FD bone, but remain unaffected for years to decades [41]. Indications for surgery include documented progressive visual disturbance, severe pain, or severe disfigurement. Surgery should be avoided in the absence of visual or hearing impairment. However, when surgery is indicated, it is very important to find a craniofacial and neurosurgical team experienced in treating craniofacial FD. It is possible the craniofacial disease may continue to progress slowly into adulthood, but this subject has not been well studied in a prospective manner [40].

There is little evidence that bisphosphonates are effective in craniofacial FD, even for pain, but in the case of pain that is difficult to manage, bisphosphonate treatment should be considered (Additional file 2).

#### 2. Axial and appendicular skeleton

The surgical management of FD has evolved over the years. While curettage and bone grafting were once considered the treatment of choice, experts in the care of FD no longer feel this is effective [31,51]. Intramedullary devices are preferable, in general [31,51]. Bracing may be helpful, but has not been well-studied. Shepherd's crook deformity can develop, sometimes very rapid, and the management in growing children is very challenging. Generally, some form of surgical intervention is recommended. Screening for and treatment of all endocrinopathies which adversely affect the skeleton should be performed [34]. This includes PP, hyperthyroidism, Cushing's syndrome, GH excess, and secondary hyperparathyroidism.

Bisphosphonates are frequently used in the treatment of FD (reviewed in [45,52]. The original thinking in the use of bisphosphonates in FD was that they would prevent the progression of disease [53]. However, more recent work concludes that bisphosphonates have no effect on the natural history of the disease [54,55]. It is clear, however, that bisphosphonates are effective in relieving bone pain associated with FD [32,45,52]. Many regimens have been suggested. The long term safety of bisphosphonates, especially in children, and the effects of relatively high doses of bisphosphonates on normal bone not affected by FD, are unknown. Therefore, to minimize exposure, our approach has been to use the minimum dose with the longest interval between doses possible needed to achieve and maintain pain relief (Additional file 2).

Maintaining the musculature around the FD bone is important for protecting the bone. Therefore, strengthening exercises and strength maintenance is important. Swimming and cycling are excellent modes of exercise for patients with FD that will promote strengthening and minimize the risk for fracture.

Scoliosis is common, and may be progressive [56]. Therefore, it is important to screen for the presence of scoliosis and monitor for any progression. Significant progression can occur within a short period of time.

### B. Endocrine

#### 1. Gonads

The management of PP in boys and girls is detailed in Additional file 3. The class of drugs with the longest history of safety and efficacy are the aromatase inhibitors [57,58]. More recently, the estrogen antagonist/agonist, tamoxifen, has also shown promise [59]. Secondary central PP in children with MAS frequently develops after a period of PP. Its manifestation is often pubertal progression in a child that had previously been well-controlled on medical therapy. The treatment of secondary central PP in children with MAS is the same as in children with idiopathic PP, and involves the use of long-acting gonadotropin releasing hormone analogues.

Ultrasound is used to screen and follow boys and men with testicular masses. These are usually Leydig tumors, but testes can demonstrate a mixture of Leydig and Sertoli cell masses. Microlithiasis is also commonly seen. The development of malignancy in the testes of men with MAS appears to be very rare. Therefore, prudent management may be observation with annual testicular ultrasounds, and CT of the chest abdomen and pelvis if there is any suspicion for malignancy. Lesions should be followed annually with ultrasound. There are no reported cases of gynecological malignancy in females with MAS.

#### 2. Thyroid

Antithyroidal medications, such as thionamides, are usually effective in controlling hyperthyroidism in MAS [60]. Spontaneous resolution of hyperthyroidism in MAS almost never occurs, so some form of definitive treatment, either in the form of surgery or radiation, is eventually indicated. Periodic ultrasounds of the thyroid to follow lesions should be performed annually. If a dominant or changing lesion is identified, fine needle aspiration is indicated to exclude cancer, given that thyroid cancer can be seen in the setting of MAS [24].

#### 3. Renal

Renal involvement, in the form of renal phosphate wasting, is seen in approximately 50% of the patients with MAS [6]. However, only some of those patients will have a degree of phosphate wasting that results in hypophosphatemia (18% of the total population seen at NIH). It is debatable whether the hypophosphatemia warrants treatment with oral phosphorus supplementation if signs of rickets are absent [44]. However, we recommend that patients with frankly low serum phosphate should be treated. Treatment is the same as that for patients with X-linked hypophosphatemia and tumor induced osteomalacia: high dose of oral phosphate with high dose of calcitriol (Additional file 1).

#### 4. Pituitary

GH excess is seen in approximately 20% of the patients with MAS [5]. GH excess has been shown to be associated with vision and hearing loss, and macrocephaly in patients with MAS [41]. For that reason, patients with GH excess and an elevated insulin-like growth factor-1 (IGF-1) should be treated. Treatment options include medications, surgery, and radiation. Effective medical treatment includes long-acting somatostatin analogues [4,5] and the GH receptor antagonist, pegvisomant [61-63]. There is greater long-term efficacy and safety with the somatostatin analogues, especially in children. Pegvisomant is significantly more expensive than the somatostatin analogues and may be associated with more side effects [61]. The goal of treatment in a growing child is to reduce the calculated serum IGF-1 Z-score to 0.

Surgery is an option, but is sometimes not possible due to the massive thickening of the skull bones. A trans-sphenoidal approach is the preferred method in pituitary surgery, but the skull base is virtually always involved with FD in patients with MAS and GH excess, so this approach is often impossible, or difficult, at best. A trans-frontal approach is a less desirable approach to the pituitary, in general, and in MAS it is also usually complicated by FD. Furthermore, in patients with MAS and GH excess the pituitary gland is almost always diffusely involved with areas of hyperplasia and adenoma (Edward Oldfield, personal communication). Therefore, the only hope for "cure" of the GH excess is a total hypophysectomy. This may be the best treatment in some cases, but physicians, surgeons, and patients should embark upon this option knowing that the patient will be rendered panhypopituitary at the end of the operation.

Radiotherapy is an option when surgery is not possible and medicinal treatment fails. However, there may be a higher risk of sarcomatous transformation [19,62].

Since most of the GH secreting tumors co-secrete prolactin [5], dopamine agonists (cabergoline, *etc*.) are usually necessary, and are effective at normalizing the serum prolactin.

#### 5. Parathyroid

While primary hyperparathyroidism has been reported to be part of the syndrome in the past [64], this may not be the case. When the mutations that cause MAS were sought in parathyroid tissue, they were not present [46]. Additionally, parathyroid hormone secretion and hyperplasia is likely not a cAMP, G_s_**α **mediated phenomenon. Secondary hyperparathyroidism due to vitamin D deficiency is common and can worsen FD [34]. Therefore, it should be screened for and treated.

#### 6. Adrenal

Cushing syndrome is one of the rarest, but most profound manifestations of MAS. In some cases, it can resolve spontaneously [9], but in most cases treatment is required, and in some cases, in spite of the best of care, it can be fatal. Adrenalectomy is probably the treatment of choice. If the child is too ill, adrenal blockade may provide stabilization of the disease and buy time until a point in time when surgery can be performed. Liver function abnormalities usually accompany neonatal Cushing syndrome, so ketoconazole as a medicinal therapy is usually not an option. Metryrapone at an initial dose of 300 mg/m^2^/day is recommended. The dose may be escalated to as high as 1200 mg/m^2^/day. In patients with a history of neonatal Cushing, adrenal reserve should be checked. Cushing syndrome in MAS is usually accompanied by hyperthyroidism, which, if not treated, can complicate and worsen the clinical course.

## Unresolved questions

While bisphosphonates are usually effective in relieving FD-related pain, whether or not the treatment of FD with bisphosphonates changes the natural history of the disease remains an open question. The most recent, and strongest data to date, suggests that they do not [54]. Ongoing placebo controlled trials in the US and Europe, should help to resolve this open question. More effective treatment for FD is needed. The development of cell based and/or G_s_**α **directed therapies may hold promise.

The best treatment for PP is also not clear. Letrozole, a potent third generation aromatase inhibitor, has recently been shown to be an effective therapy in some girls with MAS [57]. Studies suggest tamoxifen, the estrogen agonist/antagonist, may also be effective [59]. However, all trials for the treatment of PP have been uncontrolled, and this fact, combined with the extreme variability in the clinical course of the disease, makes conclusions about relative efficacy very difficult. A trial with the pure anti-estrogen, fulvestrant, in girls with MAS is underway. Controlled and head-to-head comparison trials are needed to establish the best treatment for PP in MAS.

### Support

A list of groups that support patients, families, and clinicians caring for patients with MAS is supplied in Additional file 4.

## Abbreviations

FD: fibrous dysplasia of bone; PP: precocious puberty; GNAS: G protein binding adenylyl cyclase stimulatory; cAMP: cyclic adenosine monophosphate; Gs alpha: G protein α-subunit; MAS: McCune-Albright syndrome; GH: growth hormone; NF: neurofibromatosis; NOF: Non-ossifying fibromas; CF: craniofacial; TSH: thyroid stimulating hormone; FT4: free thyroxine; T3: triiodothyronine; T4: thyroxine; FGF-23: fibroblast growth factor-23; TRP: tubular reabsorption of phosphate; TmP/GFR: maximal tubular reabsorption of phosphate per glomerular filtration rate; OGTT: oral glucose tolerance test; MRI: magnetic resonance imaging; CT: computed tomography; IGF-1: insulin-like growth factor-1; PTH: parathyroid hormone; MSH: melanocyte stimulating hormone; LH: luteinizing hormone; GHRH: growth hormone stimulating hormone; ACTH: adrenocrotical stimulating hormone; E2: estradiol; T: testosterone; 99 Tc-MDP: Technetium 99 methylene diphosphonate

## Competing interests

The authors declare that they have no competing interests.

## Consent

Written informed consent was obtained from the parents of the patients for publication of this case report and any accompanying images. A copy of the written consent is available for review by the Editor-in-Chief of this journal.

## Supplementary Material

Additional file 1 Recommendations for treatment of hypophosphatemia in MAS. This file describes the goal, treatment regimen and possible complications. Click here for file

Additional file 2 Bisphosphonate treatment. This file describes the goal, regimen and possible complications of bisphosphonate treatment. Click here for file

Additional file 3 Treatment of precocious puberty. This file describes treatment of precocious puberty in girls and boys. Click here for file

Additional file 4 Support. This file lists groups that support patients, families, and clinicians caring for patients with MAS. Click here for file
