# Supplementary material for: McCune-Albright syndrome
Source: Orphanet J Rare Dis. 2008 May 19;3:12. doi: 10.1186/1750-1172-3-12 (PMC2459161; doi:10.1186/1750-1172-3-12)
Supplement: Additional file 1 — Recommendations for treatment of hypophosphatemia in MAS. This file describes the goal, treatment regimen and possible complications. [file 1750-1172-3-12-S1.doc]

# Additional file 1

# Recommendations for treatment of hypophosphatemia in MAS

**Goal:**

Serum phosphorus in the lower limit of age-appropriate normal range

**Treatment:**

**Phosphorus**: 15-60 mg/kg/day (1-3 g/day adults), ***divided, 4-5 times daily***

Phosphorus treatment usually causes secondary hyperparathyroidism, so 1,25 vitamin D is added.

Treatment with 1,25 vitamin D not only prevents secondary hyperparathyroidism but may also increases gastrointestinal (GI) phosphorus absorption, improves bone healing (especially at high doses, and may also improve renal tubular maximum for phosphate reabsorption (*i.e.* increase TmP/GFR).

**1,25 vitamin D**: approximately 30 ng/kg/day (1.5 **μ**g/day, for a 70 kg man), range15-60 ng/kg/d. For children 0.5 **μ**g/day and 1 **μ**g/day for adults would be an appropriate dose, if there is evidence of defective mineralization.

**Possible complications:**

**Hypercalciuria**

With resultant nephrocalcinosis, nephrolithiasis and decreased creatinine clearance.

**Hypercalcemia**

Less common than hypercalciuria.

**GI upset**

Due to the phosphate. Dividing the doses over 4-5 times per day and with food helps.

**Follow-up:**

1. Baseline ultrasound to rule out nephrolithiasis (which some patients are at risk for at the outset).

2. q3 month urine (second A M void) for calcium and creatinine, if Ca/Cr ≥ 0.20, dip urine for heme, if + decrease 1,25 D, and obtain 24 hour urine for calcium and creatinine with the goal to keep urinary calcium in the normal range. If it is high, decrease 1,25 D again. If Ca/Cr ≤ 0.20 and serum phos and PTH ok, maintain regimen q3 month serum calcium, phosphorus, and PTH.
