# Supplementary material for: McCune-Albright syndrome
Source: Orphanet J Rare Dis. 2008 May 19;3:12. doi: 10.1186/1750-1172-3-12 (PMC2459161; doi:10.1186/1750-1172-3-12)
Supplement: Additional file 2 — Bisphosphonate treatment. This file describes the goal, regimen and possible complications of bisphosphonate treatment. [file 1750-1172-3-12-S2.doc]

# Appendix 2

**Bisphosphonate treatment**

##### Indication, dose, and frequency

It should be remembered that to date, the only clear indication for bisphosphonate treatment is pain relief. Therefore, relief of pain should be the clinical endpoint sought. Physicians should use the minimum dose and longest interval between doses needed to keep pain controlled. Initially, the achievement of pain control may require fairly frequent dosing. However, once pain control is achieved, the interval between doses can often be lengthened – sometimes as long as 9-12 months or more.

# Pamidronate

The greatest experience in treating FD is with pamidronate, so for that reason it may be preferred.

***Basic infusion***: after [1,2,3]: 1 mg/kg/day for 3 days, every 3 months, mixed in 1 liter normal saline, infused over 2-4 hours

Infiltration can cause a significant phlebitis; make sure the i.v. line is adequate

Bisphosphonates usually cause a flu-like response, but usually only with the first one or two infusions. Pretreatment with acetaminophen before the first infusion and around the clock during the days of infusions is recommended.

Bisphosphonates can sometimes cause hypocalcemia. This is especially true in the setting of vitamin D deficiency. Therefore, all patients should be screened for vitamin D deficiency before treatment with a bisphosphonate. In addition, additional calcium supplementation should be taken for the first several days after an infusion.

Zoledronic acid

Zoledronic acid is more potent than pamidronate, so there may be some advantages to its use. In addition, there are advantages to the dosing in terms of infused volume and time required to infuse.

***Basic infusion:*** 4 mg per dose for adults. If needed, it can be given as frequently as monthly, but this very frequent dosing should rarely be needed, and the number of infusions at this frequency should be limited.

The drug can be mixed in as little as 50 cc of normal saline, and infused over as little as 15 min. Larger volumes and slower infusion rates may decrease the acute phase reaction, which frequently occurs with the first dose.

Infiltration can cause a significant phlebitis, so make sure the i.v. line is adequate.

Zoledronic acid, like pamidronate, cause a flu-like, acute phase response, but usually only on the first one or two infusions. Pretreatment with acetaminophen before the first infusion and around the clock during the days of infusions is recommended

As with pamidronate, hypocalcemia can be a complication.

Again, pain is the primary indication for treatment and should be the clinical end point. Markers of bone metabolism will decrease, but this should not be the treatment end point.

**Oral bisphosphonates**

Oral bisphosphonates (alendronate, residronate, *etc*.) are significantly less effective than i.v. bisphosphonates, but ,may be useful in treating less severe pain. For the treatment of FD, these are used at higher doses than those used to treat osteoporosis (as in Paget’s disease). Again, pain is the clinical endpoint, and the dose should be adjusted to the minimum dose needed to relieve pain. As in Paget’s disease, the regimen is usually cycled, with several months on drug, followed by several months off drug, until pain recurs.

**Osteonecrosis of the jaw**: Osteonecrosis of the jaw has been reported in association with the use of bisphosphonates. When this occurs, it is usually in the setting of patients who have been receiving these drugs as adjuvant treatment for cancer. Patients are older, have often received very high doses of bisphosphonates, and have had previous chemotherapy treatment. No cases have been reported in patients with fibrous dysplasia.

**References:**

1. Liens D, Delmas PD, Meunier PJ: **Long-term effects of intravenous pamidronate in fibrous dysplasia of bone.** *Lancet* 1994;**343**:953-954.
2. Plotkin H, Rauch F, Zeitlin L, Munns C, Travers R, Glorieux FH: **Effect of pamidronate treatment in children with polyostotic fibrous dysplasia of bone.** *J Clin Endocrinol Metab* 2003;**88**:4569-4575.
3. Chapurlat RD, Delmas PD, Liens D, Meunier PJ: **Long-term effects of intravenous pamidronate in fibrous dysplasia of bone.** *J Bone Miner Res* 1997;**12**:1746-1752.
