# Supplementary material for: McCune-Albright syndrome
Source: Orphanet J Rare Dis. 2008 May 19;3:12. doi: 10.1186/1750-1172-3-12 (PMC2459161; doi:10.1186/1750-1172-3-12)
Supplement: Additional file 3 — Treatment of precocious puberty. This file describes treatment of precocious puberty in girls and boys. [file 1750-1172-3-12-S3.doc]

**Appendix 3**

#### Treatment of precocious puberty

Girls

While vaginal bleeding in a young child can be quite distressing for the parents and the child, the primary goal in treating PP in girls is to prevent severe short stature.

**Aromatase inhibitors**

These are the drugs that we have the longest experience with.

##### Testolactone

This is one of the first generation and less potent aromatase inhibitors. While this therapy was reported to be quite effective early on [1], subsequent studies have been less positive [2].

The dose is: 40 mg/kg/day in doses divided tid.

##### Newer aromatase inhibitors

Letrozole may be an effective treatment

The dose of **letrazole** is: 2.5 mg per day

The dose of **anastrozole** is: 1 mg per day.

**Tamoxifen**

Studies indicate that tamoxifen may be beneficial in the treatment of precocious puberty in MAS Long-term safety data are lacking, but tamoxifen does appear to be effective in slowing bone age advancement.

Boys

The goals are to prevent short stature and control the behavioral issues related to androgen excess. The medications and doses used to prevent short stature in boys (with the exception of tamoxifen) are the same as used in girls (see above). The medications used to treat the symptoms of androgen excess are:

**Spironolactone**

This is the medication with the longest record of safety and efficacy in children, and should be used first. It is usually effective.

The dose is: 5-7 mg/kg/day divided bid.

**Flutamide**

The record of safety and efficacy in children is not as long as that of spironolactone, but some clinicians feel quite comfortable using it. It has been associated with abnormal liver function tests in men with prostate cancer using higher doses. For this reason, it is prudent to check liver function tests at baseline and periodically.

The dose is: 10 mg/kg/day divided bid.

**References:**

1. **F**euillan PP, Foster CM, Pescovitz OH, Hench KD, Shawker T, Dwyer A, Malley JD, Barnes K, Loriaux DL, Cutler GB, Jr: **Treatment of precocious puberty in the McCune-Albright syndrome with the aromatase inhibitor testolactone.** *N Engl J Med* 1986;**315**:1115-1119.
2. Feuillan PP, Jones J, Cutler GB, Jr: **Long-term testolactone therapy for precocious puberty in girls with the McCune-Albright syndrome.** *J Clin Endocrinol Metab* 1993;**77**:647-651.
