# Supplementary material for: McCune-Albright syndrome
Source: Orphanet J Rare Dis. 2008 May 19;3:12. doi: 10.1186/1750-1172-3-12 (PMC2459161; doi:10.1186/1750-1172-3-12)
Supplement: Additional file 4 — Support. This file lists groups that support patients, families, and clinicians caring for patients with MAS. [file 1750-1172-3-12-S4.doc]

**Additional file 4**

# Support

Groups that support patients, families, and clinicians caring for patients with MAS include the online fibrous dysplasia support group (<http://fdsol.org/>), the Magic Foundation (<http://www.magicfoundation.org/>), and the Pagets Foundation (<http://www.paget.org/>). Information on studies at the NIH on MAS, as well as a significant library of free articles on FD and MAS can be found at the website for the Craniofacial and Skeletal Diseases Branch (<http://csdb.nidcr.nih.gov/csdb/>). The National Library of Medicine search engine PubMed offers a way to access abstracts and research articles on FD, MAS, and other diseases (<http://www.ncbi.nlm.nih.gov/PubMed/>).
